# Hyperpolarised magnetic resonance spectroscopy with lab-on-a-chip disease models

**DOI:** 10.1242/dmm.052817

**Published:** 2026-07-08

**Authors:** Irene Marco-Rius, Pernille Rose Jensen, Lotte Bonde Bertelsen

**Affiliations:** ^1^ Molecular Imaging for Precision Medicine Group, Institute for Bioengineering of Catalonia, Barcelona Institute for Science and Technology, 08028 Barcelona, Spain; ^2^ Center for Hyperpolarization in Magnetic Resonance, Department of Health Technology, Technical University of Denmark, 2800 Lyngby, Denmark; ^3^ The MR Research Centre, Department of Clinical Medicine, Aarhus University, 8200 Aarhus, Denmark

**Keywords:** Organ-on-a-chip, Microfluidic cell culture, *In vitro* modelling, Organoids, Hyperpolarised magnetic resonance spectroscopy, Metabolic flux analysis, Molecular imaging, Metabolic phenotyping

## Abstract

Hyperpolarised magnetic resonance spectroscopy (HP-MRS) enables real-time, non-invasive assessment of metabolism by increasing signal sensitivity by more than four orders of magnitude compared with conventional magnetic resonance spectroscopy (MRS). This signal enhancement is achieved by preparing nuclear spin populations in a non-equilibrium state prior to measurement, generating a substrate with a transient strongly amplified signal that enables detection of rapid metabolic conversion in real time. Integration of HP-MRS with cell-based microfluidic disease models (engineered systems in which living cells are cultured within controlled microscale environments that mimic key aspects of tissue physiology) enables dynamic metabolic profiling in physiologically relevant settings. These models are typically implemented as organ-on-a-chip platforms, where microfabricated channels enable precise control over perfusion, nutrient delivery, oxygenation and cellular interactions. Combined HP-MRS and chip-based microfluidic platforms enable direct, non-destructive assessment of metabolic transformations, with applications in biomarker discovery, treatment response assessment and personalised medicine. This At a Glance article reviews recent advances in chip-based microfluidic systems that are integrated with HP-MRS platforms, including designs compatible with benchtop and high-field nuclear magnetic resonance systems together with clinical magnetic resonance imaging systems. Key challenges for this technology include constraints imposed by signal decay time, variability in polarisation levels, injection reproducibility and the lack of standardised data processing.

## Teaser

**Summary:** Hyperpolarised magnetic resonance spectroscopy combined with microfluidic cell-based models allows metabolism measurement in real time in intact, physiologically relevant systems, enabling dynamic readouts for biomarker discovery and treatment assessment.

**Figure:**
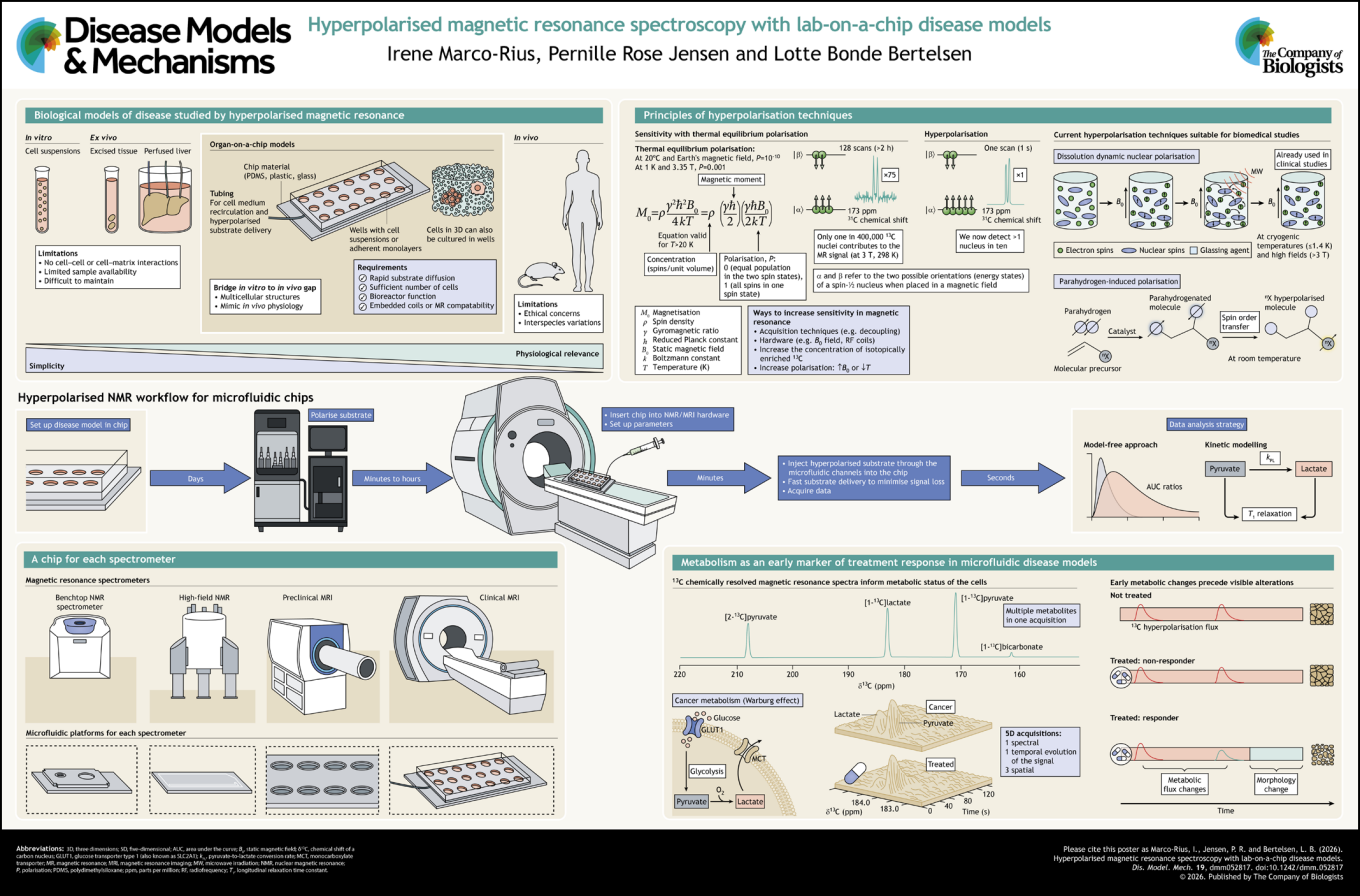
See supplementary information for a high-resolution version of the poster.

## Introduction

The discovery and validation of robust biomarkers remain critical challenges in biomedical research, particularly for improving disease diagnosis, prognosis and assessment of treatment response. Despite major advances in molecular biology, cell engineering and imaging, the translation of preclinical findings into clinical benefit remains limited. A key reason is that many experimental models used during early discovery do not adequately reproduce human physiology.

Animal models provide whole-organism complexity, but their predictive value is constrained by interspecies differences in metabolism, immune regulation, drug handling and disease progression (Guillouzo and Guguen-Guillouzo, 2008). Static human-derived cell culture systems, including Transwell^®^ inserts, spheroids and other three-dimensional (3D) models, provide important biological information, but often lack the architecture, perfusion, mechanical cues and functional complexity of native tissues (Sontheimer-Phelps et al., 2019). These limitations have driven the development of organ-on-a-chip (OoC) systems, i.e. microfluidic platforms designed to reproduce selected aspects of tissue physiology within experimentally controlled environments (Ingber, 2022) (see poster, ‘Biological models of disease studied by hyperpolarised magnetic resonance’).

Through controlled fluid flow, OoC systems can reproduce features that are difficult to achieve in static cultures, including continuous perfusion, nutrient and oxygen gradients, waste removal, shear stress and spatial organisation of multiple cell types. In other words, the flow introduces controlled transport and temporal control over the cellular microenvironment. This is particularly important when the biological question depends on dynamic processes, such as nutrient uptake, drug exposure, metabolic adaptation or stress response.

A central challenge, however, is not only reproducing tissue physiology, but measuring function dynamically within these systems. Cellular metabolism is especially attractive in this context because metabolic alterations often precede detectable changes in morphology, viability or tissue structure. Consequently, metabolism provides sensitive functional information about cellular adaptation, disease progression and therapeutic response in conditions such as cancer, liver disease and immune dysfunction (Serkova and Eckhardt, 2016; Dinzi et al., 2023; Brindle, 2024).

Magnetic resonance (MR) spectroscopy (MRS; Box 1) is a non-destructive analytical tool with high chemical specificity for studying metabolism in intact biological samples. When combined with stable isotope labelling, MRS can follow the fate of selected substrates through defined biochemical pathways. Carbon-13 (^13^C), a stable and non-radioactive isotope of carbon, is particularly useful because it is abundant in almost all metabolites. However, conventional ^13^C-MRS remains fundamentally limited by low sensitivity, especially in small-volume living systems in which metabolite concentrations are low and in cases when dynamic measurements are required.

This sensitivity barrier is now being addressed through hyperpolarisation techniques, which transiently amplify MR signals far beyond equilibrium level, resulting in signal enhancements of more than four orders of magnitude (Ardenkjaer-Larsen et al., 2003; Timm et al., 2016; Marco-Rius et al., 2021). This signal gain enables the detection of low-abundance metabolites and their rapid interconversion, making it possible to study metabolic processes over seconds to minutes. For example, [1-^13^C]pyruvate can be used to monitor downstream metabolic conversion in real time. Importantly, this provides functional information about metabolic pathway activity rather than inferring the activity indirectly from steady-state metabolite levels. Although several strategies have been developed to improve signal in ^13^C-MRS, including isotope enrichment, high-field magnets, cryogenic probes and advanced radiofrequency (RF) coil designs (Box 1), hyperpolarisation provides by far the largest signal enhancement. This unique sensitivity gain enables real-time observation of metabolic reactions across biological scales, from *in vitro* cellular systems to *in vivo* preclinical and clinical studies.

The integration of OoC systems with hyperpolarised (HP)-MRS is therefore conceptually powerful. OoC platforms provide controlled, dynamic and biologically representative environments, whereas HP-MRS enables non-invasive observation of rapid metabolic processes with high chemical specificity. Together, these technologies offer the possibility of studying functional metabolic phenotypes in systems that are more experimentally controlled than animal models and more physiologically informative than conventional static cultures.

At the same time, integration remains technically demanding. The transient nature of the hyperpolarised signal imposes strict constraints on substrate delivery, fluid handling, timing and data acquisition, whereas microfluidic devices must remain compatible with MR hardware, RF detection and reproducible biological operation. As a result, substantial engineering development has been required to adapt microfluidic systems for HP-MRS applications. This article discusses the principles underlying hyperpolarisation techniques, the range of OoC platforms developed for HP-MRS integration, and the emerging biological applications and translational opportunities enabled by these combined technologies.

Box 1. What makes a MRS experiment work Magnetic resonance spectroscopy (MRS) probes metabolism by detecting signals from nuclear magnetic resonance (NMR)-active nuclei in a strong magnetic field. In biological systems, the most used isotopes are ^1^H, ^13^C and ^31^P, which have non-zero spin and magnetic moments, which enables their detection.

The experiment is built from a few key elements that work together:

- ***B*_0_ magnetic field:** a strong, highly homogeneous magnetic field aligns nuclear spins and defines their resonance frequency. In conventional MRS, higher and more stable *B*_0_ magnetic fields increase both sensitivity and spectral resolution. In hyperpolarised (HP)-MRS, spectral resolution still benefits from a higher and more stable *B*_0_, but the large signal enhancement is generated by the hyperpolarisation method itself rather than by the acquisition *B*_0_. As a result, HP-MRS can retain high sensitivity even at lower *B*_0_.
- **Radiofrequency (RF) coil:** the RF coil delivers short RF pulses to excite the spins and then detects the signal they emit as they relax. In practice, it is both the ‘antenna’ that perturbs the system and the sensor that reads it out.
- **Pulse sequence:** a precisely timed series of RF pulses and delays that determines how spins are excited and how the signal evolves. This is where most of the experimental design sits, balancing sensitivity, temporal resolution and spectral information.
- **Gradients (for localisation):** magnetic field gradients are used when spatial information is needed (e.g. in magnetic resonance spectroscopic imaging), allowing spectra to be assigned to specific regions or voxels.
- **Signal detection and processing:** the raw signal (free induction decay) is recorded in the time domain and is Fourier transformed into a spectrum. Peak positions report on chemical environment, whereas peak intensities reflect metabolite levels.
- **Sample handling:** for biological studies, maintaining controlled conditions (temperature, perfusion, viability) of the *in vitro* systems is critical, particularly for dynamic, real-time metabolic measurements in intact *in vitro* systems. In contrast, conventional MRS-based metabolomics typically rely on cell lysis and extraction of intracellular metabolites, providing a static snapshot of metabolism at a specific time point.

## Principles of hyperpolarisation techniques

Hyperpolarisation techniques address the intrinsic sensitivity limitations of conventional ^13^C-MRS by transiently generating nuclear spin populations far from thermal equilibrium, thereby dramatically increasing the observable MR signal (see poster, ‘Principles of hyperpolarisation techniques’). In biomedical applications, the two most widely used strategies to produce liquid-state hyperpolarised metabolites are dissolution dynamic nuclear polarisation (dDNP) and parahydrogen-based methods (Stewart and Matsumoto, 2021), including parahydrogen-induced polarisation (PHIP) and signal amplification by reversible exchange (SABRE).

Among these approaches, dDNP is currently the most established hyperpolarisation method for biomedical HP-MRS, spanning applications from *in vitro* cell studies to clinical imaging (Larson et al., 2024). In dDNP, polarisation is transferred from unpaired electron spins to nuclei by microwave irradiation at cryogenic temperatures (∼1 K) and high magnetic fields (∼3-7 T) (see poster, ‘Principles of hyperpolarisation techniques’). The polarised solid sample is then rapidly dissolved to produce an injectable or perfusable solution that can be delivered to cells, tissues or living organisms for MRS detection (Marco-Rius and Comment, 2018).

Parahydrogen-based techniques use the singlet spin order of parahydrogen gas as the source of hyperpolarisation (see poster, ‘Principles of hyperpolarisation techniques’). In PHIP, spin order is transferred through chemical hydrogenation of an unsaturated precursor, whereas in SABRE, polarisation transfer occurs through reversible binding of the substrate and parahydrogen to a metal catalyst, typically an iridium-based complex, without permanent chemical modification of the substrate. Compared with dDNP, these approaches can generate hyperpolarised molecules rapidly at or near room temperature without requiring cryogenic instrumentation, making them attractive for faster and potentially more accessible workflows (Cavallari et al., 2018; Knecht et al., 2021). However, substrate scope, catalyst compatibility, purification requirements and biological applicability remain important practical limitations.

Other hyperpolarisation approaches, including brute-force polarisation, Overhauser dynamic nuclear polarisation and spin-exchange optical pumping have also been explored (Levien et al., 2024; Hirsch et al., 2015; Nikolaou et al., 2014), although they have been less widely applied to studies of living cell systems.

A fundamental limitation shared by all hyperpolarisation approaches is that the enhanced signal is transient. Hyperpolarised magnetisation decays according to the longitudinal relaxation time (*T*_1_), which typically ranges from seconds to minutes depending on the nucleus, molecular structure, magnetic field strength (*B*_0_) and physicochemical environment. Consequently, the biologically useful observation window is inherently limited and imposes strict timing constraints on substrate preparation, transfer, delivery and acquisition (see poster, ‘Principles of hyperpolarisation techniques’).

This transient and non-renewable nature of hyperpolarised signal also requires dedicated acquisition strategies. Conventional MRS sequences designed for thermal equilibrium are often inefficient because each RF excitation consumes a significant part of the available polarisation. HP-MRS, in contrast, commonly uses rapid spectroscopic imaging methods to provide spatial and spectral information within the short lifetime of the hyperpolarised state (Gordon et al., 2021; Oerther and Marco-Rius, 2023).

In most implementations, hyperpolarisation is generated in a dedicated instrument called a polariser, which should ideally be placed in immediate proximity to the nuclear MR (NMR) spectrometer or MR imaging (MRI) scanner to minimise transfer delays and polarisation loss. Nevertheless, advances in compact polarisers, automated handling systems and *in situ* substrate preparation are progressively improving the accessibility and scalability of HP-MRS workflows (Capozzi et al., 2017, 2026; Ji et al., 2017). These developments are particularly relevant for microfluidic systems, in which precise synchronisation between polarisation, substrate delivery, flow control and MRS acquisition is critical.

## Microfluidic OoC platforms for integration with HP-MRS

OoC systems provide a versatile framework for studying *in vitro* cell metabolism within controlled, more physiologically relevant environments. These platforms span a broad range of biological complexity, from simple perfused chambers containing two-dimensional (2D) cell layers to multi-compartment devices that reproduce tissue–tissue interfaces, vascular perfusion or inter-organ communication (Sung et al., 2019). The appropriate degree of complexity depends strongly on the biological question being addressed. Although the microfluidic platforms vary, a fairly standard workflow has emerged (Box 2).

In the context of HP-MRS, commonly implemented microfluidic configurations include: (1) single-chamber microfluidic devices containing 2D or 3D cell cultures, (2) multi-well platforms enabling parallel measurements and (3) interconnected systems designed to reproduce organ-level interactions under continuous flow (see poster, ‘A chip for each spectrometer’).

These platforms support diverse biological models, including adherent monolayers, spheroids, hydrogel- or cryogel-based scaffolds and engineered tissue constructs within microlitre-to-millilitre-scale volumes compatible with MRS detection. Because hyperpolarisation is transient, device architectures must support rapid and reproducible substrate delivery while minimising dead volume, dispersion and transfer delays. Controlled perfusion also helps maintain stable environmental conditions during acquisition, thereby reducing variability associated with fluctuations in oxygenation, nutrient availability and waste accumulation.

The integration of microfluidics with HP-MRS imposes several engineering constraints. Device geometries must remain compatible with RF coil sensitivity profiles while minimising inactive sample volume. Materials must be MRS compatible and avoid magnetic susceptibility artefacts. Fluidic architectures must additionally support reproducible timing and efficient delivery within the finite lifetime of the hyperpolarised signal. These constraints directly influence channel dimensions, tubing length, flow rates, mixing strategies and cell positioning relative to the RF-sensitive region.

As a result, a range of tailored microfluidic platforms has emerged. Traditional MRS setups employ cylindrical RF coils optimised for homogenous excitation magnetic fields (*B*_1_) and relatively large sample volumes. In contrast, the high sensitivity and negligible background signal of HP--MRS enables miniaturised or geometry-adapted coil designs that can be directly coupled to microfluidic platforms (Jeong et al., 2017; Christensen et al., 2024, 2025b; Azagra et al., 2026). Planar microfluidic chips are typically coupled with tailored surface RF coils, enabling localised detection. This can be done either as a single sample or as multi-well platforms that are designed for higher throughput (Patra et al., 2021; Mathiassen et al., 2026; Rogers et al., 2023; Christensen et al., 2024, 2025b; Azagra et al., 2026). Other approaches focus on compatibility with existing NMR or MRI hardware, including inserts adapted to standard NMR tubes (Lauritsen et al., 2015; Mangas-Florencio et al., 2025; Mathiassen et al., 2023) or preclinical and clinical MRI volume RF coils (Yeste et al., 2023).

Each configuration involves trade-offs between sensitivity, throughput, biological complexity, ease of fabrication and compatibility with different MRS systems. Consequently, no single platform architecture is currently optimal for all biological or translational applications. Despite substantial progress, important limitations remain. The integration of OoC systems with HP-MRS is technically demanding and often requires bespoke hardware, limiting accessibility and standardisation across laboratories. Biological reproducibility also remains challenging, particularly in 3D cultures in which cell density, scaffold architecture, diffusion barriers and long-term viability can vary between experiments (Temple et al., 2022; Azagra et al., 2024). Moreover, although OoC systems capture selected aspects of tissue physiology, they remain simplified models that may lack full cellular diversity, immune components, vascular and neural regulation, or long-term homeostatic stability. Improving robustness, scalability and standardisation therefore remains a major priority for the field.

Box 2. Workflow and timeline of a HP-MRS experiment with microfluidic chips The integration of hyperpolarised magnetic resonance spectroscopy (HP-MRS) with organ-on-a-chip systems requires precise coordination between biological preparation, device engineering, polarisation chemistry, substrate delivery and magnetic resonance (MR) acquisition. Although individual platforms differ, most workflows follow a common experimental sequence designed to preserve polarisation and extract metabolic information within a short acquisition window (see poster, ‘Hyperpolarised NMR workflow for microfluidic chips'):

1. **Preparation of the biological model (days to weeks):** cells may be cultured as two-dimensional monolayers, spheroids, scaffold-embedded constructs, etc. Once the desired cell density, differentiation state, treatment condition or disease phenotype has been established, the biological construct is loaded into the microfluidic chip, unless it has been cultured directly within the device. The chip must maintain viability, support perfusion and remain compatible with the MR hardware.
2. **Hyperpolarised substrate production (minutes to hours):** on the day of the HP-MRS experiment, a metabolic substrate is hyperpolarised in a polariser, boosting the signal far beyond conventional levels. Dissolution dynamic nuclear polarisation typically requires tens of minutes to hours to reach high polarisation levels (>50%) (Christensen et al., 2025a), whereas parahydrogen-induced polarisation is achieved within minutes, with polarisation levels typically exceeding ∼15% (Stewart and Matsumoto, 2021).
3. **Chip positioning inside the MR system (minutes):** while the substrate is being polarised, the organ-on-a-chip is positioned inside the MR system and aligned with a radiofrequency coil or probe adapted to the sample geometry. Depending on the application, this may involve a benchtop nuclear MR (NMR) spectrometer, a high-field NMR system, a preclinical MR imaging (MRI) scanner or a clinical MRI system. The choice of platform determines the available sensitivity, spectral resolution, spatial encoding capability and throughput.
4. **Hyperpolarised substrate transfer (seconds):** the hyperpolarised substrate is then rapidly transferred to the chip through a dedicated injection or perfusion line. Because the enhanced signal begins to decay immediately upon leaving the polariser, governed by the intrinsic *T*_1_ relaxation time, timing is critical. The arrival of the substrate at the cells must be synchronised with the start of MR acquisition.
5. **Data acquisition (seconds to minutes) and quantification:** once the substrate reaches the biological sample, dynamic MR spectra or spectroscopic images are acquired. The resulting time series captures the formation of downstream metabolites. Data are processed to extract metabolite ratios, area-under-the-curve (AUC) values or kinetic parameters. Model-free approaches, such as metabolite or AUC ratios, provide robust summary metrics that are useful for comparison across conditions. Model-based approaches, such as two-site or multi-site exchange models, can estimate apparent conversion rates and provide more mechanistic information, although they require careful assumptions regarding substrate delivery, relaxation, transport and radiofrequency excitation.

## Metabolism as an early marker of disease progression and treatment response in microfluidic disease models

Metabolic alterations are increasingly recognised as early and sensitive indicators of disease progression and therapeutic response. *In vivo* imaging studies using positron emission tomography (PET), proton (^1^H)-MRS and ^13^C-MRS have shown that metabolic changes often precede anatomical or morphological alterations in diseases such as cancer (Meng et al., 2023), liver disease (Diniz et al., 2020) and neurodegeneration (Shivamurthy et al., 2015). In the context of drug development and personalised medicine, early metabolic readouts can provide crucial insights into therapeutic efficacy before conventional imaging techniques detect structural effects (Brindle, 2024).

HP-MRS is particularly powerful for characterising real-time metabolic conversion (i.e. fluxes) in key cellular pathways, including glycolysis and mitochondrial metabolism. Unlike endpoint metabolomics approaches, which measure steady-state metabolite abundance, HP-MRS provides kinetic information about how labelled carbon moves through active biochemical pathways in real time. Unlike PET, which primarily reports tracer uptake and retention, HP-MRS resolves multiple downstream products from a single labelled substrate. Hyperpolarised [1-^13^C]pyruvate is the most widely used example (see poster, ‘Metabolism as an early marker of treatment response in microfluidic disease models’).

The conversion of pyruvate into lactate, alanine and bicarbonate provides information about cytosolic redox balance, amino acid metabolism and mitochondrial oxidative metabolism, respectively. Importantly, interpretation remains dependent on transport kinetics, substrate delivery and cellular context. In this way, HP-MRS provides pathway-specific, quantitative insight into metabolic processes in intact systems (Sriram et al., 2015).

Cancer metabolism illustrates the value of this approach. Many tumours exhibit increased pyruvate-to-lactate conversion despite oxygen availability, consistent with aerobic glycolysis or the Warburg effect (see poster, ‘Metabolism as an early marker of treatment response in microfluidic disease models’). HP-MRS captures this directly as an increased lactate-to-pyruvate flux, often accompanied by reduced bicarbonate production. These measurements can be sensitive to changes in cell viability, redox balance, transporter activity and therapeutic response – in some cases before conventional viability assays or anatomical imaging reveal measurable effects.

The translation of this concept into *in vitro* disease models has progressed through several experimental formats. Most HP-MRS studies in living cells have historically been performed in relatively simple systems, including cell suspensions (Sannelli et al., 2023a; Meier et al., 2024), packed cells (Sriram et al., 2018), spheroids in NMR tubes (Tee et al., 2018), bioreactors or perfused chambers (Nielsen et al., 2016), rather than fully integrated OoC platforms. Although these systems were essential for demonstrating feasibility, they often provided limited control over tissue architecture, flow dynamics and multicellular organisation.

More recently, advances across microfluidics and MRS technologies have expanded the range of compatible metabolic models. Conventional ^1^H-MRS metabolomics have been implemented in microlitre-scale biofluids using 2D microfluidic chips for rapid metabolic profiling (Hale et al., 2018; Sharma and Utz, 2019; Rogers et al., 2023). Moreover, metabolomics have been combined with HP-MRS to achieve enhanced sensitivity in biomarker detection for prostate cancer in adenocarcinomas of the mouse prostate (Frahm et al., 2021) and for immortalised human cell lines (Frahm et al., 2020; Lerche et al., 2018). HP-MRS has been used to monitor real-time metabolic fluxes, such as pyruvate-to-lactate conversion, in cell suspensions and spheroids using custom fabricated microcoils (Jeong et al., 2017; Lees et al., 2021). In parallel, bioreactors incorporating 3D scaffolds, hydrogels and continuous flow have been developed to study tumour metabolism, drug response and nutrient dynamics using both conventional and HP-MRS approaches (Lauritsen et al., 2015; Mathiassen et al., 2023; Mangas-Florencio et al., 2025). Complementary in-cell NMR strategies have extended this approach to live-cell environments, revealing how substrate mixtures influence intracellular pathway flexibility, with implications for both drug efficacy and drug resistance mechanisms (Sannelli et al., 2023a,b). Together, these studies show that dynamic metabolic flux analysis can reveal adaptive treatment responses that may be missed by static or endpoint assays.

The major opportunity now lies in combining the metabolic readouts with more biomimetic disease models. OoC systems can reproduce defined features of the disease microenvironment, including hypoxia, nutrient gradients, matrix composition, perfusion and tissue–tissue interfaces. When integrated with HP-MRS, these systems could help determine how environmental conditions influence metabolic adaptation and treatment sensitivity. This is particularly relevant in cancer, metabolic liver disease and immunometabolism, in which cellular function is strongly shaped by nutrient availability, oxygenation and intercellular communication.

Patient-derived systems represent another important translational direction. Organoids, tumour explants and patient-derived cells are increasingly used to capture individual variability in disease biology and treatment response. However, these models are often limited by sample availability, heterogeneity and the need for non-destructive readouts. HP-MRS could provide dynamic metabolic information from limited material while preserving the sample for longitudinal analysis or complementary endpoint assays.

Interpretation of HP-MRS data, however, requires careful biochemical contextualisation. Changes in labelled lactate production, for example, should not be interpreted solely as alterations in glycolytic activity. They may also reflect changes in pyruvate transport, lactate dehydrogenase activity, NADH/NAD^+^ balance, intracellular substrate availability, cell viability or extracellular metabolite pools. Similarly, bicarbonate production reflects pyruvate dehydrogenase activity, but it is also influenced by substrate delivery, mitochondrial function and cellular energetic state. For this reason, HP-MRS measurements are most informative when integrated with orthogonal approaches such as metabolomics, enzymatic analysis, transcriptomics, microscopy or viability measurements.

## Future perspectives

For HP-MRS-compatible microfluidic platforms to move beyond proof-of-concept demonstrations, the field must now prioritise reproducibility, standardisation and context-of-use.

Standardisation will likely require harmonised workflows for biological preparation, perfusion conditions, substrate delivery, hyperpolarised substrate quality control, acquisition design and data analysis. Automated fluid handling and synchronised substrate injection may reduce operator-dependent variability and improve experimental reproducibility. Parallelised multi-sample platforms could further improve comparability by enabling several biological conditions to be analysed during the same polarisation event.

It will also be important to define where HP-MRS-compatible microfluidics provide clear advantages relative to alternative technologies. Static 3D cultures, spheroids in NMR tubes, bulk cell suspensions, scaffold-based bioreactors and conventional metabolomics may remain preferable when throughput, simplicity or broad metabolic coverage are prioritised. Fluorescence-based metabolic sensors offer high spatial and temporal resolution but are generally limited to selected pathways and may require exogenous probes or genetic modification (Sadoine et al., 2021). HP-MRS occupies a distinct position within this analytical landscape by providing chemically specific, non-invasive and time-resolved measurements of metabolic fluxes in intact living systems.

Broader adoption will depend on improving accessibility and transferability. Commercial or semi-standardised hyperpolarisation-compatible chip platforms could reduce the need for bespoke engineering, whereas compact and automated polarisation systems may expand access beyond specialised centres. Integration with complementary approaches, including fluorescence microscopy, transcriptomics, proteomics and endpoint metabolomics, may provide a more comprehensive understanding of cellular state and therapeutic response.

Ultimately, the long-term impact of HP-MRS-compatible microfluidic platforms will depend not only on demonstrating technical feasibility, but also on establishing robust and biologically validated workflows capable of supporting meaningful pharmacological or clinical decision making.

In conclusion, the convergence of HP-MRS and microfluidic disease models is shifting metabolic analysis from endpoint biochemistry towards functional phenotyping. OoC systems provide controlled and increasingly biomimetic environments, whereas HP-MRS enables direct observation of metabolic pathway activity with high chemical specificity. The next phase of the field will require a transition from bespoke proof-of-concept studies towards reproducible, scalable and standardised platforms. If these challenges can be addressed, HP-MRS-compatible OoC systems may provide a uniquely powerful framework for studying treatment response and metabolic adaptation in human-derived models under experimentally controlled conditions.

## Poster

Poster
