# Supplementary figures and images for: Hyperpolarised magnetic resonance spectroscopy with lab-on-a-chip disease models
Source: Dis Model Mech. 2026 Jul 8;19(6):dmm052817. doi: 10.1242/dmm.052817 (PMC13382991; doi:10.1242/dmm.052817)

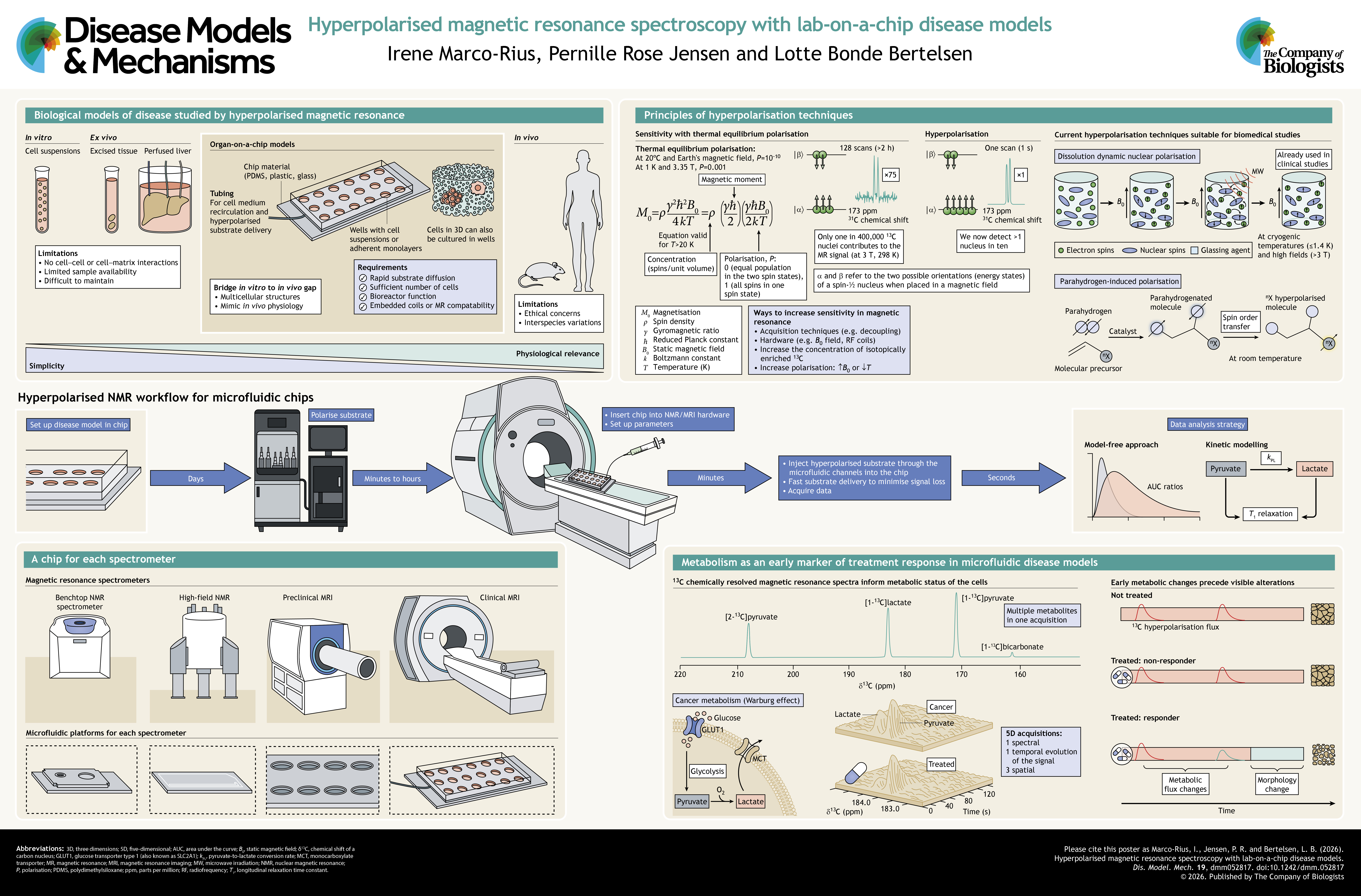

Supplement: Poster [file dmm-19-052817-s1.jpg]
